# Marked variations in the effectiveness of commercial disinfectants against clinically relevant antibiotic-resistant bacteria and fungi

**DOI:** 10.2478/aiht-2026-77-4065

**Published:** 2026-06-30

**Authors:** Josip Figl, Stjepan Nemec, Zrinka Bošnjak, Ana Budimir, Tomislav Ivanković

**Affiliations:** University Hospital Centre Zagreb, Department of Surgery, Zagreb, Croatia; University of Zagreb Faculty of Science, Department of Biology, Zagreb, Croatia; University Hospital Centre Zagreb, Department of Clinical and Molecular Microbiology, Zagreb, Croatia

**Keywords:** *Acinetobacter baumannii*, alcohols, *Candida albicans*, carbapenem-resistance, chlorhexidine, *Enterococcus faecium*, hospital-acquired infections, hydrogen peroxide, *Klebsiella pneumoniae*, minimal bactericidal concentration, noncritical surfaces, quaternary ammonium compounds, *Staphylococcus aureus*, *Acinetobacter baumannii*, alkoholi, bolničke infekcije, *Candida albicans*, *Enterococcus faecium*, *Klebsiella pneumoniae*, klorheksidin, kvaterni amonijevi spojevi, minimalna baktericidna koncentracija, nekritične površine, otpornost na karbapeneme, peroksidi, *Staphylococcus aureus*

## Abstract

Effective disinfection of noncritical hospital surfaces may be essential for limiting the transmission of antibiotic-resistant bacteria (ARB), which seem to persist despite clear disinfection guidelines and raise concern about the potency of real-world disinfectants and about the consistency of disinfection practices. This study compared the bactericidal efficacy of eight commercially available disinfectants – alcohols, hydrogen peroxide, quaternary ammonium compounds (QACs), and chlorhexidine (CHX) – against multidrug-resistant clinical isolates of *Klebsiella pneumoniae*, *Acinetobacter baumannii*, *Staphylococcus aureus*, *Enterococcus faecium*, carbapenem-resistant *Pseudomonas aeruginosa*, and model organism *Candida albicans* as the most frequent causes of hospital-acquired infections in Croatia. Several formulations, particularly those based on QACs and CHX, retained full bactericidal activity even when diluted 10–32 times after only 1 min of contact, while alcohols were ineffective when diluted more than twice. However, all the formulations were effective when applied at full concentration. These findings highlight marked differences in the intrinsic bactericidal strength of commercial products, especially if not applied properly, which may explain variable outcomes observed in routine hospital disinfection practices. These issues may be overcome by rotating different disinfectant classes during routine surface decontamination and by optimising disinfectant strength to improve ARB and fungal control in clinical settings.

## Abstract (hr)

Učinkovita dezinfekcija nekritičnih bolničkih površina mogla bi biti ključan korak u smanjenju brojnosti bakterija otpornih na antibiotike (ARB) u zdravstvenim institucijama. Unatoč jasnim smjernicama za dezinfekciju, ove su bakterije i dalje prisutne u bolničkom okolišu, što izaziva zabrinutost glede učinkovitosti i dosljednosti dezinfekcijske prakse. Ovdje predstavljena studija usporedila je baktericidnu učinkovitost osam komercijalno dostupnih dezinficijenasa iz skupine alkohola, vodikova peroksida, kvaternih amonijevih spojeva (QAC) i klorheksidina (CHX), prema višestruko otpornim bolničkim izolatima bakterija *Klebsiella pneumoniae*, *Acinetobacter baumannii*, *Staphylococcus aureus*, *Enterococcus faecium* i *Pseudomonas aeruginosa* te prema gljivici *Candida albicans* kao najčešćih uzročnika bolničkih infekcija u Hrvatskoj. Spojevi na bazi QAC-ova i CHX-a, zadržali su punu baktericidnu aktivnost čak i kada su bili razrijeđeni 10 – 32 puta te nakon samo jedne minute kontakta. Alkoholi su, na primjer, izgubili učinkovitost kada su bili razrijeđeni više od dva puta. No, treba naglasiti da su sve formulacije bile potpuno učinkovite kada su se primjenjivale u punoj, preporučenoj koncentraciji. Rezultati upućuju na značajne razlike u baktericidnoj snazi komercijalnih proizvoda, posebice ako se ne primjenjuju pravilno, što može objasniti prisutnost bakterija i nakon čišćenja. Predlaže se stoga rutinska izmjena različitih klasa dezinficijensâ tijekom tretiranja nekritičnih površina radi što učinkovitijeg smanjenja prijenosa bakterija otpornih na antibiotike i gljivice u bolnicama.

Considering the undisputable spread of hospital (healthcare) acquired infections (HAIs), there seems to be a disconnect between theoretical efficacy and real-world performance of disinfection practices (1, 2). While active ingredients such as quaternary ammonium compounds (QACs), alcohols, and chlorine-based agents are proven to maximally reduce microbial load in controlled conditions such as laboratory testing and validation protocols, in real life hospital surfaces remain reservoirs for pathogens, antibiotic-resistant strains in particular (3,4,5).

To clarify this issue, it is helpful to revisit the traditional Spaulding’s classification, which divides surfaces and medical instruments into three categories based on infection risk: critical, semi-critical, and noncritical. Critical items contact sterile tissues and must be sterilised. Semi-critical items touch mucous membranes and require high-level disinfection. Noncritical items, such as bed rails, overbed tables, blood pressure cuffs, door handles, and other frequently touched surfaces, contact only intact skin and are assumed to pose minimal infection risk (3, 6). For decades, noncritical surfaces were largely overlooked in infection prevention protocols. As recently as 2004, experts noted that these surfaces had not been directly implicated in disease transmission but may contribute to cross-transmission by allowing transient hand carriage by healthcare personnel or patients (7). Nowadays, a growing body of evidence shows that noncritical surfaces harbour clinically significant pathogens and serve as indirect transmission vehicles, either through hand contamination of healthcare personnel or contact with mobile medical equipment (4, 8, 9).

Persistent surface contamination remains a well-documented problem, despite the routine use of commercial disinfectants whose effectiveness has passed standard testing. This may be owed to poor compliance with disinfection protocols and reliance on visual cleanliness alone, rather than microbiological monitoring, as most hospitals do not routinely test surfaces for bacterial load unless responding to an outbreak (8, 9).

In real-life conditions, disinfectant effectiveness is often diminished. For example, instructions for all commercial formulations clearly require spraying the target surface and leaving it wet for a minimal contact time, usually 1 min, but real-life disinfection practice is to spray the surface and wipe it off almost immediately. Such deviations from recommended contact times have repeatedly been documented in routine hospital practice, largely due to workload and time constraints (3, 4, 9). It is unrealistic to expect strict compliance with all instructions at every moment under the often chaotic circumstances in clinical practice. Therefore, a more effective approach to improving hospital disinfection may be to identify real-world challenges and limitations and to seek optimal solutions that are sustainable under actual working conditions. The challenge does not seem to be the lack of effective products but ensuring their optimal use.

A wide range of disinfectants is used in healthcare settings to control surface contamination. These include quaternary ammonium compounds (QACs), alcohols, hydrogen peroxide, chlorhexidine, and phenolics, each targeting microbial membranes or intracellular structures via different mechanisms (9, 6).

In this context, we set out to gain new insights into the performance of widely used hospital disinfectants under suboptimal application conditions involving short contact times and dilutions that reflect real hospital workflows and evaluate how clinically significant and highly resistant pathogens respond to them to better understand their survival potential and to identify practical improvements for infection control in everyday hospital practice.

## MATERIALS AND METHODS

In this study, we selected eight commercial disinfectants commonly used in clinical settings across Croatia. They cover a range of biocidal classes and active ingredients, including alcohols, QACs, chlorhexidine, and oxidative compounds (6, 15, 16). The rationale behind their selection was the widespread use in hospital practice, representativeness of major disinfectant categories, and literature-supported indications of reduced susceptibility or resistance in clinically relevant bacteria (10, 11). The commercial names of the used formulations are not listed but have been submitted to the editorial office. A detailed description and composition of formulations are given in Table 1. All the formulations were originally packed, unopened prior to experimentation, and tested within the expiration date.

**Table 1:** Description and composition of disinfectant formulations used in the study

**Abbreviation**	**Active compound and its concentration**	**Recommended use**
F1Alc	Ethanol 70 %	General disinfection
F2Alc	Ethanol 57 % and isopropyl alcohol 6 %	Cleaning and disinfection of surfaces and non-invasive medical instruments
F3Alc	Isopropyl alcohol 70 %	Cleaning and disinfection of surfaces and non-invasive medical instruments
F4Oxy	Hydrogen peroxide 15 g/L	Cleaning and disinfection of invasive and non-invasive medical devices
F5Oxy	Hydrogen peroxide 15 g/L and phenoxyethanol 5 g/L	Surface cleaning and disinfection
F6QAC	Isopropyl alcohol 17 %; didecyldimethylammonium chloride 2.3 g/L	Surface disinfection
F7QAC	Alkyldimethylbenzilammonium chloride 2.6 g/L; didecylmethyl ammonium chloride 2.6 g/L; alkylethylbenzilammonium chloride 2.6 g/L	Cleaning and disinfection of surfaces and non-invasive medical instruments, including the ones sensitive to alcohol
F8CHX	Chlorhexidin gluconate 8 g/L in 80 % ethanol	Hand disinfection and hand preparation for surgery

The bacterial panel was selected based on the 2024 WHO Bacterial Priority Pathogens List (12) and national surveillance data from the Croatian Academy of Medical Sciences (13) and includes six clinical isolates of carbapenem- and extended spectrum beta-lactamase-resistant *Klebsiella pneumoniae*, carbapenem-resistant *Acinetobacter baumannii* and *Pseudomonas aeruginosa*, methicillin-resistant *Staphylococcus aureus*, and vancomycin-resistant *Enterococcus faecium*. In addition, we used model organisms, namely the bacterium *Escherichia coli* DSM 498 (Deutsche Sammlung von Mikroorganismen), *S. aureus* (ATCC 25923), and fungus *Candida albicans* (ATCC 10231). These species are frequently implicated in healthcare-associated infections and, according to the national surveillance reports, Croatia has some of the highest carbapenem-resistance rates in Europe for *K. pneumoniae* and *A. baumannii* (2, 13). The model organism *Candida albicans* was included, because *C. albicans* was the most common species isolated from hospital patients in 2023 (13). This is especially significant in the context that the first confirmed case of highly resistant *Candida auris* infection was noted in September 2025 in University Hospital Centre Split, Croatia (14).

All the isolates were gathered at the University Hospital Centre Zagreb, Croatia, during the last trimester of 2024. Detailed description and susceptibility profiles are given in Table 2. Isolates were identified by matrix-assisted laser desorption ionisation-time of flight mass spectrometry (MALDI-TOF) and antibiotic susceptibility profiled with the VITEK^®^2 system (bioMérieux, Marcy l'Étoile, France), according to the European Committee on Antimicrobial Susceptibility Testing (EUCAST) standards.

**Table 2:** Description and antibiotic susceptibility profile of clinical bacterial isolates used in the study

**Species**	**Origin**	**Resistant to:**	**Susceptible to:**
*Escherichia coli* DSM 498	Laboratory microbank	-	AMC, TZP, CAZ, CAZ-AVI, CPM, MEM, IMI, AK, CN, CIP, FOF, COL
*Staphylococcus aureus* ATCC 25923	Laboratory microbank	-	AMC, AZI, CTX, E, ETP, IMI, P
*Candida albicans* ATCC 10231	Laboratory microbank	-	AmB, AFG, MFG, CAS, FLZ
*Klebsiella pneumoniae* (carbapenem-resistant)	Urine culture	AMP, AMC, CXM, SXT, NOR, CIP, LEV, CRO, CTX, TZP, CPM, ETP, MEM, IMI	COL, CAZ-AVI
*Klebsiella pneumoniae* (extended spectrum beta-lactamase-resistant)	Faeces sample	AMP, AMC, CXM, SXT, CN, CAZ, CRO, CTX, TZP, CPM	AK, MEM
*Acinetobacter baumannii* (carbapenem-resistant)	Tracheal aspirate	CN, AK, TZP, MEM, IMI	AMS, COL
*Pseudomonas aeruginosa* (carbapenem-resistant)	Haemoculture	MEM, IMI	AK
*Staphylococcus aureus* (methicillin-resistant)	Nasopharyngeal swab	CLOX, E, AZI, CLA, CLI, CIP, MOXI, LEV	SXT, CN, RD, VAN, TEC, LZD, MUP
*Enterococcus faecium* (vancomycin-resistant)	Perianal swab	AMP, VAN, TEC	LZD

Antibiotic abbreviations: AK – amikacin; AMC – amoxicillin + clavulanic acid; AMP – ampicillin; AMS – ampicillin + sulbactam; AZI – azithromycin; CAZ – ceftazidime; CAZ-AVI – ceftazidime-avibactam; CIP – ciprofloxacin; CLA – clarithromycin; CLI – clindamycin; CLOX – cloxacillin; CN – gentamicin; COL – colistin; CPM – cefepime; CRO – ceftriaxone; CTX – cefotaxime; CXM – cefuroxime; E – erythromycin; ETP – ertapenem; FOF – fosfomycin; IMI – imipenem; LEV – levofloxacin; LZD – linezolid; MEM – meropenem; MUP – mupirocin; MOXI – moxifloxacin; NOR – norfloxacin; P – benzylpenicillin; RD – rifampicin; SXT – trimethoprim-sulfamethoxazole; TEC – teicoplanin; TZP – piperacillin-tazobactam; VAN – vancomycin. Antifungal medication abbreviations: AmB – amphotericin B; AFG – anidulafungin; MFG – micafungin; CAS – caspofungin; VOR – voriconazole; FLZ – fluconazole

### Minimum bactericidal concentration assay

Antibacterial efficacy of the selected disinfectants was tested with the minimum bactericidal concentration (MBC) assay using a modified broth microdilution method. While based on standard methodology, certain steps were adapted as described below.

Each bacterial strain was revitalised from the Microbank^™^ cryostorage system (Pro-Lab Diagnostics, Ontario, Canada) on Mueller-Hinton Agar (Biolife, Milan, Italy) prior to experimentation. From freshly grown biomass, a bacterial suspension of approximately 5×10^5^ CFU/mL was prepared in Mueller-Hinton Broth (Biolife). A volume of 0.5 mL of this suspension was then distributed into sterile 1.5 mL Eppendorf-type plastic tubes.

**Figure 1:**
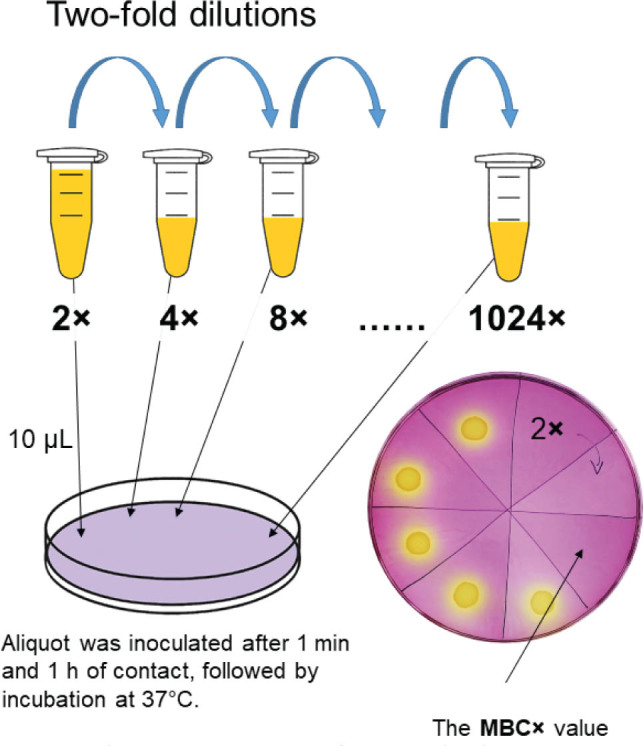
Schematic representation of MBC× value determination

In the first tube, 0.5 mL of disinfectant formulation was added, yielding a 2-fold dilution of the original disinfectant concentration. Twofold serial dilutions were then repeated, resulting in a dilution series ranging from 2× to 1024× (Figure 1).

From each tube, a 10 µL aliquot was aseptically plated onto Dey-Engley neutralising agar (NutriSelect^®^ Plus, Merck KGaA, Darmstadt, Germany) at two time points: after 1 min and 1 h of contact. Plates were incubated at 37 °C for 24 h. The highest dilution at which no visible bacterial growth was observed was recorded as the MBC× value (Figure 1).

The key distinction between this and standard MBC assays lies in the interpretation: in this assay, a higher MBC× value (i.e., a greater dilution factor) indicates greater disinfectant efficacy, as the agent remains bactericidal even when substantially diluted. In contrast, in standard MBC protocols – where the dilution series begins with the highest concentration – a lower MBC value corresponds to stronger efficacy, as less compound is required to achieve complete bacterial eradication.

**Figure 2:**
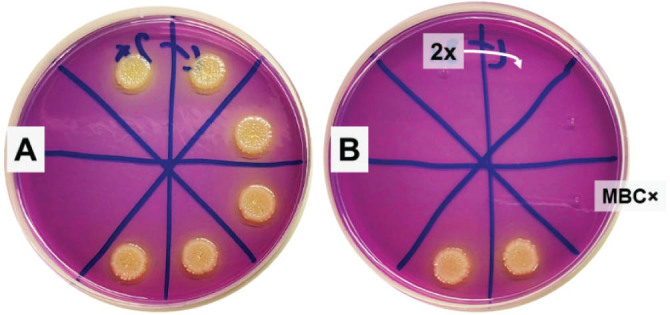
Dey-Engley agar neutralisation experiment. On plate A, 5 µL of the disinfectant at the corresponding concentration was first inoculated. After 1 min, 5 µL of the bacterial suspension was added to the same spot. On plate B, a standard MBC assay was performed, showing the MBC× value at a 16× dilution after 1 min of contact. If the agar effectively neutralised the disinfectant, bacteria from the suspension should grow without inhibition, as shown in the figure above. The images presented correspond to *K. pneumoniae*/F5Oxy testing

Neutralising agar was used to terminate the activity of disinfectants after 1 min and prevent prolonged antimicrobial action and false positive results. The Dey-Engley medium contains a broad-spectrum combination of neutralisers, including sodium thiosulphate, sodium bisulphite, sodium thioglycolate, lecithin, and polysorbate 80, which effectively inactivate the disinfectants used in the experiments. Even though the efficacy of the Dey-Engley agar was evidenced in multiple publications (17,18,19,20), we tested its neutralisation efficacy as shown in Figure 2.

All the experiments were done in biological triplicate.

## RESULTS

When applied in full concentration all tested formulations eradicated all bacterial cells (initial inoculum ~10^5^ CFU/mL) in suspension after just one minute of contact time. Even the twofold dilutions remained completely effective, in line with previous studies demonstrating high efficacy of commercial disinfectants against clinically relevant bacteria in suspension tests (21, 22).

However, further dilutions revealed significant differences in the strength of various formulation types. In this context, the term “stronger” refers to disinfectants that exhibited minimum bactericidal concentrations (MBC× values) at higher dilution factors (lower concentrations).

**Figure 3:**
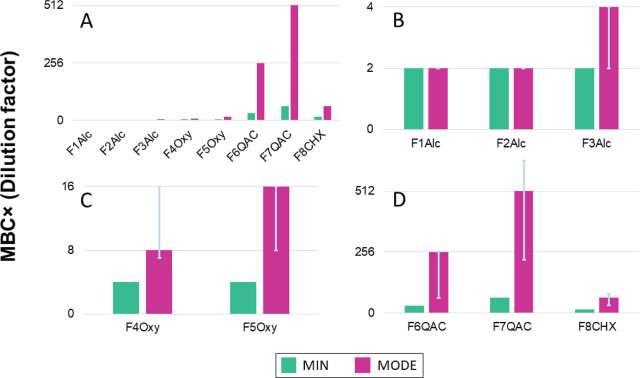
Mode and minimal MBC× values for all the tested bacterial strains combined, expressed as dilution factors of tested disinfectants after 1 min of contact. A) all the tested formulations; B) alcohols; C) peroxides; D) quaternary ammonium compounds and chlorhexidine. Error bars represent the interquartile range (Q1–Q3) of mode values

When all MBC× values after 1 min of contact and across tested bacterial strains are considered, the two QAC-based formulations stand out as particularly effective (Figure 3), with respective mode MBC× values (the value that appears most frequently in a data set) of 256 and 512.

In general, alcohol-based formulations were the least effective, followed by peroxide-based formulations, then chlorhexidine, and finally QACs. Evaluating the lowest effective dilution factors (minimum MBC× after 1 min of contact) across all tested bacteria (Figure 3), we found that alcohol formulations retained effectiveness at a 2-fold dilution. Both peroxide-based formulations, F4Oxy and F5Oxy, were effective at 4-fold dilutions. The chlorhexidine formulation remained effective at a 16-fold dilution, while the QAC-based formulations, F6QAC and F7QAC, retained full efficacy at 32- and 64-fold dilutions, respectively. The QAC formulations show a discrepancy between the recorded mode and minimum values (Figure 3A and D), primarily due to the inclusion of carbapenem-resistant *P. aeruginosa*, which is known to exhibit exceptionally high intrinsic resilience to the action of quaternary ammonium compounds (23, 24).

**Figure 4:**
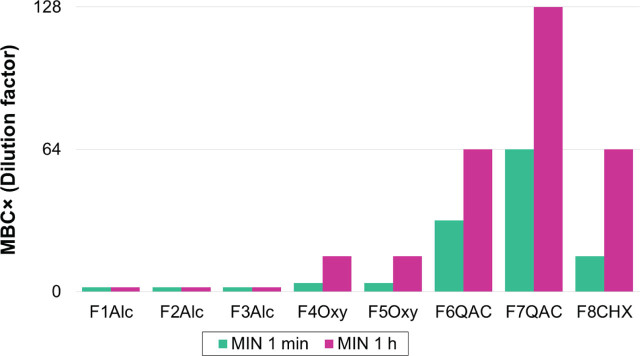
Minimum MBC× values for all bacterial strains, expressed as dilution factor of disinfectants after 1 m and 1 h of contact

As expected, increasing contact time generally enhanced the efficacy of most formulations, with the exception of alcohol-based disinfectants, which remained effective only at a two-fold dilution and showed no improvement over time (Figure 4). In contrast, other disinfectants demonstrated two to four times better performance at 1 h contact compared to 1 min. In other words, contact time is an essential feature for all but alcohol-based disinfectants.

**Figure 5:**
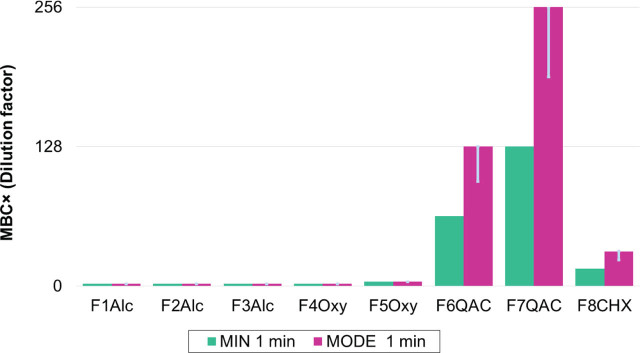
Mode and minimal MBC× values for *C. albicans*, expressed as dilution factor of disinfectants after 1 min of contact. Error bars represent the interquartile range (Q1–Q3) of mode values

The results for *C. albicans* showed a similar pattern, with QAC formulations being much stronger (Figure 5).

**Figure 6:**
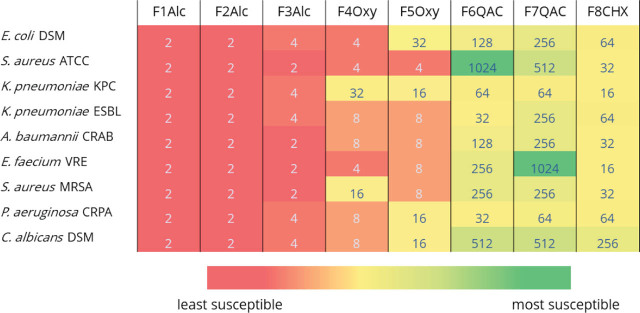
Heat map of disinfectant efficacy against the tested strains based on the minimum MBC× values after 1 min of contact (also shown as numerical values). Red tones denote more resilient strains requiring more concentrated formulations for bactericidal effect, whereas green tones indicate more susceptible strains, for which highly diluted formulations remain bactericidal

Figure 6 compares the tested species based on their minimal MBC× values after 1 min of contact. Alcohol- and chlorhexidine-based formulations demonstrated relatively uniform efficacy, with no marked interspecies differences. The exceptions are *P. aeruginosa* and *K. pneumoniae*, which turned out to be more resistant to QACs. On the other hand, the vancomycin-resistant *E. faecium* and *S. aureus* ATCC strain were far more susceptible to QACs than other strains.

## DISCUSSION AND CONCLUSION

It should be noted that the small number of isolates tested per species does not allow for a robust quantitative comparison, but the observed differences may still be indicative of species-related susceptibility patterns.

Building on previous studies comparing the efficacy of different classes of disinfectants against multidrug-resistant isolates (25, 26), our study provides additional practical insights into which formulations exhibit the strongest and weakest bactericidal strength.

Our findings are consistent with previous research showing that commonly used formulations including alcohols, QACs, hydrogen peroxide, and chlorhexidine exhibit rapid bactericidal activity in suspension tests when used correctly and at recommended concentrations (15, 16, 22, 27). However, in real-life applications their performance can vary greatly due to multiple factors, such as organic load, surface type, dilution errors, and insufficient contact time (6).

In an attempt to mimic realistic hospital environment, it was crucial to employ testing methods that reflect bactericidal potential under realistic conditions as accurately as possible. This is why we opted for MBC measurements over the standard minimum inhibitory concentration (MIC), which merely indicates the lowest concentration that inhibits visible growth and does not equate to actual microbial death. In contrast, the minimum bactericidal concentration (MBC) determines the lowest concentration that kills ≥99.9 % of bacterial cells and is more appropriate for disinfection studies, especially when the goal is total eradication (10, 11).

In addition to selecting MBC over MIC, our study focused on a 1-minute contact time, which reflects realistic surface disinfection scenarios in hospital settings. Current international guidelines (6, 29) highlight that short contact times are standard in routine disinfection protocols, especially for noncritical surfaces in general wards. Testing disinfectants under these stringent conditions allows for meaningful comparisons of their immediate efficacy, avoiding overestimation of performance based on prolonged exposure. Furthermore, Ni et al. (11) have demonstrated that while many commercial disinfectants appear highly effective in extended contact assays, significant variation in bactericidal activity becomes apparent under reduced contact times, especially against resistant strains such as carbapenem-resistant *K. pneumoniae*.

Standardised disinfectant efficacy testing, such as ISO EN 13727, evaluates disinfectants at concentrations of 80 % or less and includes the addition of interfering substances, such as bovine serum albumin (0.3 g/L or 3 g/L), to simulate organic load under defined clean or dirty conditions. In contrast, the MBC× values presented here started from 50 % disinfectant concentration and were determined in Mueller-Hinton broth, a complex, nutrient-rich medium containing approximately 21 g/L of proteins and other organic components, which exceeds the organic load used in standardised disinfectant testing protocols and therefore constitutes a more rigorous test environment for bactericidal activity than ISO EN 13727.

Among the tested bacterial strains, *P. aeruginosa* and *K. pneumoniae* stood out by demonstrating markedly lower susceptibility to QAC-based disinfectants. This finding aligns with previous reports highlighting *P. aeruginosa* as a species intrinsically less susceptible to QACs due to its robust outer membrane, capacity for efflux pump overexpression, and biofilm formation (23, 24). In our study both the lowest and most variable MBC× values were associated with the carbapenem-resistant *P. aeruginosa* strain, suggesting a complex interplay between resistance to antibiotics and disinfectants. Similar patterns have been observed by Pottier et al. (30), who reported that 28 % of *P. aeruginosa* strains isolated over a ten-year hospital sur veillance period exhibited lower susceptibility to didecyldimethylammonium chloride (30). In addition, some hospital-adapted *P. aeruginosa* strains have been shown to withstand benzalkonium chloride concentrations of 1200–1600 µg/mL, which are higher than the concentrations of many commercial products (24). A similar trend of higher resistance to QACs was reported for *K. pneumoniae* (31), not just in clinical settings but also in a natural environment, indicating intrinsic resistance (32).

Our results suggest that while most tested formulations are effective against a broad spectrum of bacteria, QAC-based disinfectants require particular scrutiny when used against *P. aeruginosa* and *K. pneumoniae*, especially in settings where antibiotic resistance is also present.

They also call for more stringent and evidence-based disinfection practices for noncritical hospital surfaces. Although commercial disinfectants undoubtedly meet regulatory standards, our findings demonstrate considerable variation in efficacy across different formulations and bacterial species, particularly at reduced contact times or when diluted. This supports the growing consensus that even noncritical surfaces can act as reservoirs for clinically significant and potentially multidrug-resistant pathogens (29, 33).

In Croatia, national cleaning standards for hospitals established in 2018 (34) clearly define procedural requirements and visual cleanliness criteria but they lack microbiological performance benchmarks such as acceptable CFU/cm^2^ levels or reduction thresholds following disinfection. Similarly, the UK National Specifications for Cleanliness include structured visual audit systems but do not mandate microbiological monitoring (8). The CDC guidelines stress the role of routine disinfection but acknowledge that there is no universally accepted microbiological threshold for “clean” surfaces, especially in general wards (6). In this context, our data provide a rationale for updating institutional disinfection protocols and incorporating routine efficacy testing and risk-based rotation of disinfectants.

We recommend that hospitals should consider alternating disinfectants based on their potency and spectrum of activity, periodically replacing more powerful agents (e.g. QACs or chlorhexidine) with less aggressive ones. As for the question of why not simply use the most potent disinfectant at all times, identified as QACs in our study, continuous and exclusive use may promote tolerance development, select for more resilient strains, and lead to residue accumulation on surfaces, potentially affecting cleaning performance and antimicrobial susceptibility (15, 16, 28, 30). The rotation strategy, already established in pharmaceutical and food industries, may reduce the selective pressure for cross-resistance while maintaining hygiene standards (10). Additionally, targeted reinforcement of surface disinfection, especially in high-touch and near-patient zones, should be prioritised in line with both CDC recommendations and evolving European guidelines (2, 29, 35). Ultimately, our findings support the need for continuous quality improvement in environmental hygiene, not only through product selection but also through standardised protocols, staff training, and systematic re-evaluation of disinfectant effectiveness under real-life conditions.
